# Technical note: Recommendations for a standard procedure to assess cortical bone at the tissue-level in vivo using impact microindentation

**DOI:** 10.1016/j.bonr.2016.07.004

**Published:** 2016-07-26

**Authors:** A. Diez-Perez, M.L. Bouxsein, E.F. Eriksen, S. Khosla, J.S. Nyman, S. Papapoulos, S.Y. Tang

**Affiliations:** a Department of Internal Medicine, Hospital del Mar-IMIM, Autonomous University of Barcelona and RETICEF, Instituto Carlos III, Spain; b Center for Advanced Orthopedic Studies, Beth Isreal Deaconess Medical Center and Department of Orthopedic Surgery, Harvard Medical School, Boston, MA, USA; c Dept. of Endocrinology, Morbid Obesity and Preventive Medicine, Oslo University Hospital, Oslo, Norway; d Division of Endocrinology and Kogod Center on Aging, Mayo Clinic, Rochester, MN, USA; e Department of Orthopaedic Surgery and Rehabilitation, Vanderbilt University Medical Center, Nashville, TN, USA; f Center for Bone Quality, Leiden University Medical Center, Leiden, The Netherlands; g Department of Orthopaedic Surgery, Washington University in St Louis, St Louis, MO, USA

**Keywords:** Bone microindentation, Impact microindentation, Bone tissue characteristics

## Abstract

Impact microindentation is a novel method for measuring the resistance of cortical bone to indentation in patients. Clinical use of a handheld impact microindentation technique is expanding, highlighting the need to standardize the measurement technique. Here, we describe a detailed standard operation procedure to improve the consistency and comparability of the measurements across centers.

## Highlights

• A protocol for impact microindentation in clinics is described.
• Measurements can be standardized by adopting a common protocol.
• The aim of the work is to minimize the inter-center variability.
• This protocol reflects the experience of the 7 involved centers.

## 1 Introduction

Microindentation has emerged as a novel technique for the measurement of tissue-level material properties of cortical bone. Unlike traditional microindentation involving angled tips with sharp corners (e.g., Rockwell, Vickers, Berkovich), extensive surface preparation is not necessary with the new microindentation technology for bone assessment called Reference Point Indentation (RPI). RPI devices currently available for research utilize different methods of microindentation: 1) cyclic microindentation; and 2) impact microindentation, whereby the “micro” denotes the length scale of the indentation. Both approaches indent bone using a stainless steel probe with a spheroconical tip (2.5 μm or 10 μm radius, respectively). The former technique measures the force vs. displacement response of bone tissue to indentation via cyclic loading and unloading between 0 N and up to 10 N at 2 Hz. Although cyclic microindentation was used in living humans when the technology was initially developed (Diez-Perez et al., 2010, Guerri-Fernandez et al., 2013), currently it is primarily used for laboratory testing of ex vivo samples or animals (BioDent® Reference Point Indenter, Active Life Scientific, Santa Barbara, CA, USA). The latter method, impact microindentation, is now utilized in living humans, and is performed using a hand-held RPI device (OsteoProbe® RUO Reference Point Indenter, Active Life Scientific, Santa Barbara, CA, USA) that imparts a single impact load to the bone surface. The handheld OsteoProbe is more amenable for clinical use than the BioDent, which requires a stand to perform measurements in human patients (Randall et al., 2013). Both techniques have been recently reviewed (Allen et al., 2015).

These indentation methods have previously been referred to by different names. Here we propose the use of impact microindentation (IMI) to describe the indentation method for the clinical device (Osteoprobe RUO) and cyclic reference point microindentation (CMI) for the laboratory device (BioDent), in order to unify the nomenclature going forward.

Since the use of both devices is expanding, there is a critical need for standard measurement procedures in order to optimize consistency across different centers and thereby permit multicenter comparisons. For this reason, and since the use of IMI in clinical studies is growing (Farr et al., 2014, Malgo et al., 2015, Duarte Sosa et al., 2015, Mellibovsky et al., 2015, Rudang et al., 2015, Sundh et al., 2015), a group of investigators with direct experience in their use proposes a standardized procedure detailed herein.

## 2 Materials and methods

### 2.1 Methodology of the measurement

**Fig. 1:**
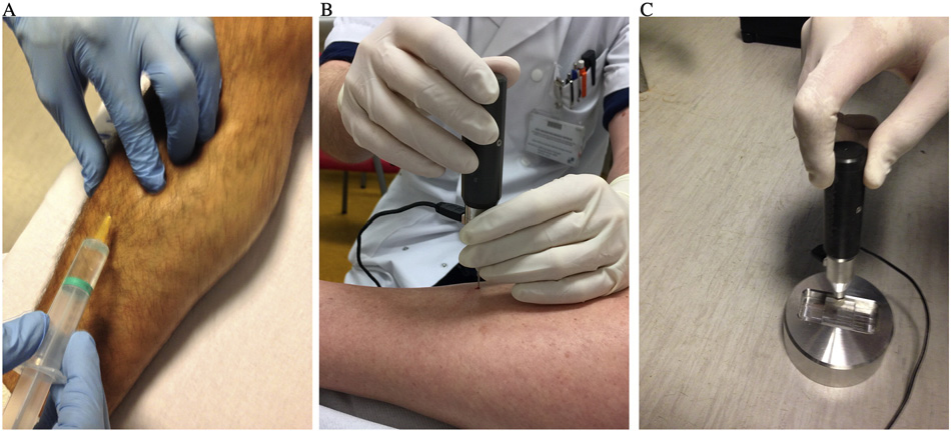
A. Infiltration with subcutaneous local anesthesia; B. Piercing with the test probe until reaching the periosteum. Then the probe must be placed perpendicular to the bone surface; C. Indentation in the BMSi-100 Reference Material, also keeping perpendicularity to the surface and at the same speed as in the tibia. See text for details.

A detailed step-by-step description of the measurement procedure is hereby explained. The experience of the different centers of the co-authors is the common basis for the protocol measurement we propose. Fig. 1 A–C depicts how the procedure is performed in real patient measurements.

1. Position the patient in decubitus supine position for better comfort. The non-dominant tibia is selected for the measurement unless some local contraindication is present (see below), in which case the contralateral side can be used.
2. Position the leg in external rotation to orient the flat surface of the medial tibia diaphysis horizontal (i.e., parallel to the exam table).
3. Mark the mid distance between the medial border of the tibia plateau and the medial malleolus using a measuring tape.
4. Perform a careful disinfection of a wide area of the anterior mid-tibia region using a chlorhexidine solution or any other local disinfectant.
5. Perform local anesthesia infiltration by inserting a thin syringe needle both subcutaneously and in the periosteal surface. Lidocaine 2%, mepivacaine 2% or equivalent, with or without adrenaline, can be used.
6. Place the BMSi-100 reference material cube, clamped within the standard holder, on a firm surface.
7. While local anesthesia is taking over, the operator, ideally assisted by another person that operates the computer, wears sterile gloves after hand washing or disinfecting with a topical solution. The operator, for an optimal procedure, remains blinded to the computer screen.
8. Insert a sterile probe into the Osteoprobe.
9. Pierce the skin and periosteum at the marked mid diaphysis point of the medial tibia, until reaching the bone cortex.
10. Without losing probe contact with the bone surface, adjust the angle of the device to become perpendicular to the tibia surface, with a variation degree inferior to 10°, and slide the outer housing of the device toward the patient's leg to initiate a measurement (see below for details).
11. For every indentation, the body of the device is pulled down slowly and smoothly for a 2 to 3 s period.
12. The first measurement should be systematically disregarded since there is often inadequate penetration of the probe through the periosteum.
13. After the measurement, slide the probe to a new location at least 2 mm away from the prior measurement, re-adjust the angulation of the device, and perform another measurement. Do this until 8–10 measurements are obtained without pulling the probe out of the skin.
14. Remove those results considered as not valid (see below for discussion of invalid results). At least 5 valid measurements must be obtained to consider the procedure acceptable.
15. After the set of measurements in the bone have been completed and the invalid measurements removed, the normalization phase starts following the software indications, by indenting eight times in the BMSi-100 Reference Material, also keeping perpendicularity to the surface and at the same speed as in the tibia.
16. The screen will then display the result as the bone material strength index (BMSi).

### 2.2 Some technical considerations to be taken into account

**Fig. 2:**
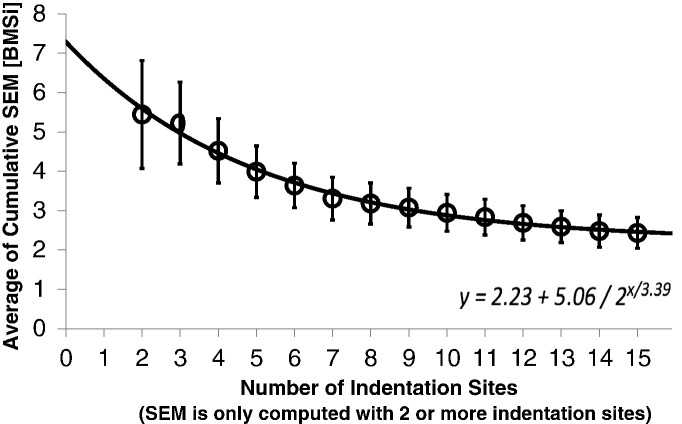
By examining the average cumulative variability of increasing number of indentation sites, we can optimize for the number of indentations needed to converge toward the minimum variation. Standard error of the mean, a statistic for the standard deviation of the estimated population mean, is computed for successive indentations, e.g. SEM of the first two indentations, then SEM of the first 3 indentations, etc. These SEMs are then averaged over a 30 unique subjects to determine the average variation for each subject as a function of the number of indentations. As the indentation numbers increase, the variability is reduced and thus the likelihood of an outlier measurement skewing the mean is also reduced. The exponential fit of this data suggests a value BMSi value of 2.23 is unavoidable in this cohort. At 8–10 indentations, the average SEM value ranges from 3.18 to 2.94 BMSi units, and this should provide magnitude of the detectable difference in this population.
• The measurements can be obtained in two parallel lines of five indentations each, following the longitudinal axis of the tibial diaphysis, although measurements in a circle may also be performed. Usually the elasticity of the skin permits relocation of the probe without removing the probe from the initial skin piercing. Close proximity to the borders of the tibial diaphysis should be avoided.
• Once the ten measurements are completed, those considered not valid should be removed. Currently, there is no automated system incorporated to the software to remove invalid measurements. Thus, as of today, we consider that a measurement can be considered invalid if: 1) The measurement is flagged by the software because it lies outside the limits of the “green zone” provided by the software; or 2) When the observer notices that the “texture” of the indented bone is grossly abnormal (e.g., like indenting “cork “).
• On occasion, before starting the actual indentation, the device captures a false signal and a grossly abnormal value is shown in the screen and should be removed. The decision of removing a measurement should be taken in real time, ideally by a second observer to minimize potential bias.
• BMSi, Bone Material Strength index, is defined as 100 times the harmonic mean of indentation distance increase from impact (IDI) into a standard calibration material, H(IDI(BMSi-100)), divided by the IDI into the bone, IDI (Bone).
• When the skin is fragile (i.e. older individual or in patients on glucocorticoids) sometimes a second or a third piercing is performed to avoid a scratch in the skin, to complete the set of ten measurements. Each time the skin is pierced anew the first measurement should be discarded. Likewise, in young individuals with tough skin, two to four points of skin piercing may be necessary to prevent the probe from suffering lateral tension by the skin itself that might interfere with the measurement.
• For longitudinal measurements the procedure should be repeated using the same protocol. The same tibia is indented and in order to ensure that the measured area is comparable with previous measurements, the location of the mid diaphysis of the tibia should be carefully targeted.
• It is recommended that a new probe be used for every measurement (i.e., between patients) since otherwise the precision of the technique can be affected because the interaction conditions between the base of the tip and the mechanisms of the device may be modified.
• Careful training is crucial to ensure reliable measurements. Training should include indentations on reference materials (BMSi-73 & BMSi-100) until consistently achieving values of 100 ± 1.5 for BMSi-100 and 73 ± 2.0 for BMSi-73, with standard deviations < 1.5 for both. Then, training in cadavers is advisable. All this process should be done under close supervision of an experienced user.

• Every indentation applies a preload force of 10 N at which point a trigger system releases a mechanism that applies an additional 30 N force at high speed, making a microscopic indent in the cortex of the tibia.
• Since perpendicularity to the tibia flat surface is critical, it is advisable that, ideally, an assistant can help by taking a different angle of view.
• The need for taking eight to ten measurements is based on the minimal variability achieved (Fig. 2) taking into account that the bone surface has some intrinsic irregularities.

### 2.3 Contraindications for the procedure

The contraindications for making impact microindentation include local or systemic problems that potentially could interfere with the technique or constitute a risk for the patient (Table 1). Patients on anticoagulants can be measured.

**Table 1:** Contraindications for impact microindentation.

Local edema^a^
Local skin infection or cellulitis^a^
Prior clinical or stress fracture in the tibia diaphysis^a^
Dermatological lesions in the area of measurement^a^
Focal tibial lesions like in primary or metastatic tumor, Paget's disease, Gaucher, etc.^a^
Osteomyelitis of the tibia^a^
Systemic infection or fever (unless unrelated to infection)
Severe obesity
Any other condition in the opinion of the operator
Allergy to lidocaine or alternative local anesthetic used

a In these cases, the contralateral tibia, if free of the problem, can be used.

To date, based on the experience of the authors of this manuscript, only two complications from over > 1300 procedures have been observed; one case of mild anaphylactic reaction to local anesthetic that was treated with diphenhydramine and resolved; and one case of mild skin infection in a kidney transplant recipient that quickly resolved with antibiotics. No local pain, bone damage or other issue in the indented tibia has been observed.

## 3 Discussion

We describe a common protocol followed in the authors' centers for impact microindentation measurements in clinical patients. This way we aim to improve the reproducibility of the test when performed by different observers and/or at different centers.

Bone mineral density (BMD) is a strong, independent predictor of bone propensity to fracture (i.e. resistance to trauma) in the average patient, most notably in postmenopausal osteoporosis. However, there are clinical situations where bone resistance to trauma is not well explained by bone density levels and, also, most of the low-trauma fractures occur in individuals with BMD levels above the osteoporosis threshold. Therefore, other components besides density likely play a role in the capability of bone to absorb energy and resist a traumatic impact without fracturing. The recent development of bone microindentation has opened the possibility of testing the ability of bone to resist the impact of a tiny probe in creating microscopic cracks in the surface of cortical bone. Several clinical experiments have shown worse microindentation properties in groups of patients as type 2 diabetes mellitus (Farr et al., 2014), fracture in patients with osteopenia (Malgo et al., 2015), HIV-infected individuals (Guerri-Fernandez et al., 2016), or glucocorticoid-treated patients (Mellibovsky et al., 2015) in spite of a relatively preserved or normal BMD. Therefore the technique captures some of the non-density components of bone strength with the advantage of potentially being applicable in clinical practice.

## 4 Possible determinants of BMSi

The impact microindentation measurement is a unique mechanical assessment of bone because it involves a micron-size tip engaging the tissue at a very high loading rate (Table 2).

**Table 2:** Differences in tips and loading rate among indentation techniques.

Characteristic	OsteoProbe (IMI)	BioDent (cRPI)	Nanoindentation
Indenter shape	90° spheroconical	90° spheroconical	Berkovich^a^
Material of test probe	Stainless-steel	Stainless-steel	Diamond
Radius of indenter tip	10 μm	2.5 μm	–
Nominal indent size^b^	350 μm	200 μm	5 μm
Maximum force	40 N	10 N	0.03 N
Approximate indentation depth	150 μm–260 μm	30 μm–70 μm	0.1–1 μm
Time interval of loading	0.25 ms	167 ms^c^	100 ms
Effective loading rate	120,000 N/s	60 N/s	0.3 N/s

a Berkovich, akin to 3-sided pyramid, is the most widely used tip geometry in the nanoindentation of bone, but spheroconical tips can be accommodated.

b Diameter of indenter (IMI and cRPI) and edge of tip (nanoindentation) as observed by SEM.

c With cRPI, there are two cycles of load-dwell-unload in 1 s (2 Hz).

Identification of the characteristics of cortical bone that influence BMSi is an active area of research, and the clinically important determinants of BMSi have yet to be identified. Given the size of the indenter tip, and knowledge about the hierarchical organization of bone's constituents at different length scales (Rho et al., 1998), it is possible to surmise what bone features may influence the measurement. Thus, BMSi likely depends on a number of factors that could affect the resistance of the tissue to IMI including: i) the primary collagen fibril orientation relative to the indent direction (axial vs. transverse orientation), ii) the cross-linking profile of collagen I (proportion of immature to mature crosslinks as well as the amount and type of glycation-mediated, non-enzymatic crosslinks), iii) the relative amount of mineral to matrix (degree of mineralization), and iv) the number of interlamellar interfaces (potential for sliding as dictated by mineral-collagen interactions).

Furthermore, bone is a viscoelastic-viscoplastic material, whose elastic (e.g., modulus) and post-yield behaviors are dependent on the rate of loading. The effect of loading rate on IMI or CMI measurements is not known, but typically in load-to-fracture tests of cortical bone, the post-yield energy dissipation (Hansen et al., 2008) and resistance to crack growth (Zimmermann et al., 2014) decrease with increasing strain rate, while elastic modulus increases. Thus, although BMSi measured by IMI may correlate with traditional mechanical properties of bone at the apparent level (independent of macrostructure but not microstructure), the mode of deformation and the resulting time-dependent mechanical behavior of the tissue is fundamentally different between IMI and bending, tensile-compression-torsion, or fracture toughness tests which are conducted at slow (i.e., quasi-static) loading rates. Note that tissue-level properties derived from quasi-static microindentation (1.67 × 10^− 3^ N/s) do not correlate with mechanical properties of human cortical bone derived from quasi-static tests at the apparent level (Mirzaali et al., 2015) likely because apparent-level properties also reflect microstructural features such as porosity and osteonal area.

Since the age-related loss of cortical bone typically occurs near the endosteal surface of long bones (Zebaze et al., 2010, Perilli et al., in press) distant from the indentation sites at the periosteal surface, the chance of indenting regions with relatively large pores (> 100 μm) is small. Any resorption spaces or Haversian/Volkmann's canals near the indentation site would lower BMSi as bone tissue could be pushed into the void spaces. To minimize the potential influence of these surface cortical pores on BMSi, multiple IMI measurements are made, thereby reducing the influence of a single measurement that may be near a pore.

**Fig. 3:**
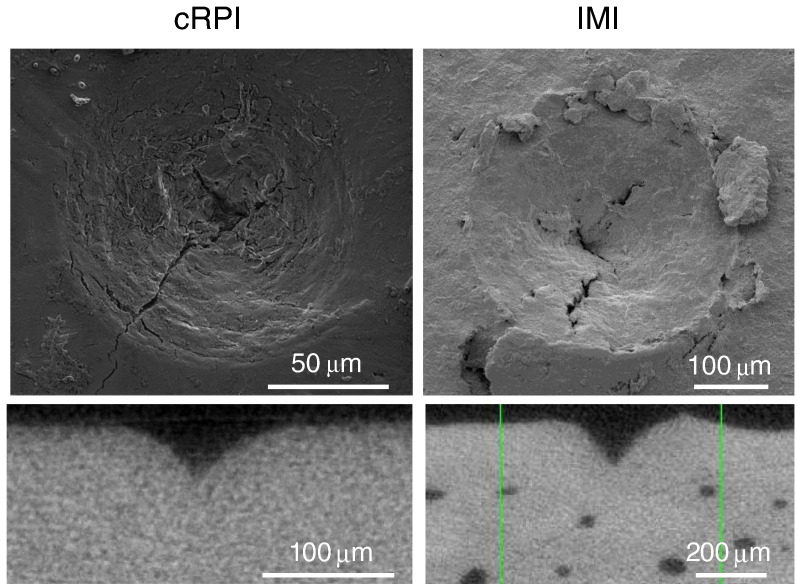
SEM (top) and μCT (bottom) images of indents from cRPI and IMI. Microcracks are visible in indent region by SEM suggesting damage formation and propagation is involved. The depth of the indent is higher for IMI than for cRPI.

**Fig. 4:**
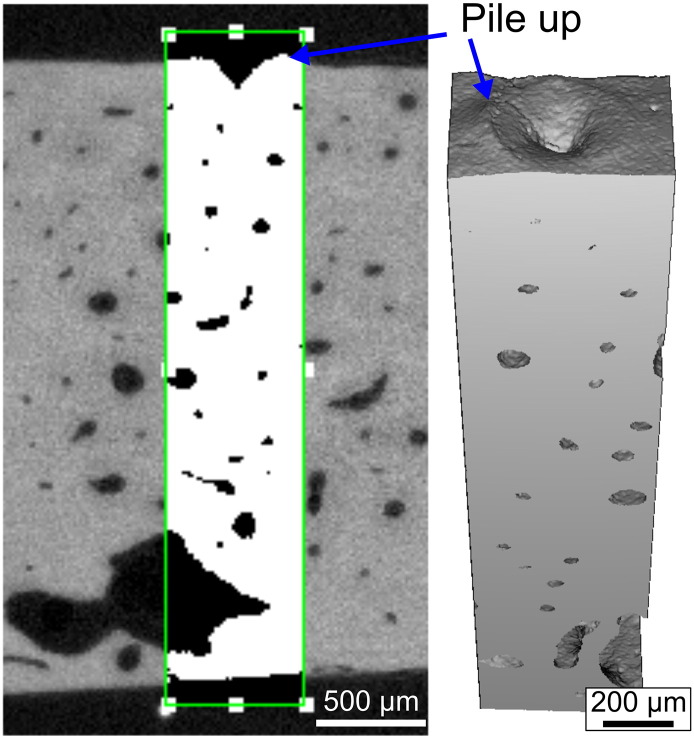
High μCT rendering of an indents from IMI on a cadaveric tibia mid-shaft. There is evidence of bone tissue piling-up above the surface.

Scanning electron microscopy (SEM) images of indents from CMI and IMI (Fig. 3) have revealed the presence of microcracks emanating from the indentation site suggesting BMSi depends on the ability of tissue to resist crack initiation and growth (i.e., fracture toughness). This is certainly a possibility, but confirming the impact of these toughening mechanisms on the BMSi measurement itself is rather difficult due to the challenge of quantifying crack propagation near the probe tip during IMI. Pile-up of the tissue at the surface can also occur during IMI of hydrated cadaveric tibia without surrounding soft tissue (Fig. 4) suggesting that the tissue undergoes plastic flow as the OsteoProbe tip indents the bone.

Findings from a recent finite element simulation of an OsteoProbe indentation of equine cortical bone (Hoffseth et al., 2015) and nanoindentation of ovine cortical bone with smaller 90°. spheroconical tip (radius = 0.6 μm) (Mullins et al., 2009) suggest that yielding of the tissue below the tip is pressure dependent and plastic deformation involves frictional mechanisms. Thus, these simulations indicate that asymmetric yield occurs during indentation as well the separation lamellar interfaces illustrating the complexities of the indentation modality.

In summary, there is still much to be learned about the age- and disease-related factors including the specific tissue composition and nanostructural attributes that influence BMSi. Nonetheless, this mechanical measurement of indentation resistance is unique and may provide additional information about bones' mechanical behavior beyond the better-known mechanical properties of bone such as strength and toughness. Thus, although considerable work needs to be done to better understand exactly what properties of bone BMSi represents, defining this should proceed in parallel with studies to better define the potential clinical utility of this measurement. While delineating the biomechanical basis of the BMSi measurement is clearly important from a scientific perspective, the future of this technology rests ultimately with whether BMSi will provide information beyond current fracture risk assessment tools (e.g., DXA and FRAX) in terms of better identifying patients at increased fracture risk.

## Conflict of interest

AD-P owns shares of Active Life Scientific, the manufacturer of the impact microindentation device. All other authors declare no conflict of interest.
